# Enhancing Adherence in Cognitive Assessment and Training: Insights From the APPT Trial

**DOI:** 10.1093/geroni/igaf122.301

**Published:** 2025-12-31

**Authors:** Dawn Carr, Walter Boot, Zhe He, Shayok Chakraborty, Mia Liza Lustria, Shenghao Zhang, Michael Dieciuc

**Affiliations:** Florida State University, Tallahassee, Florida, United States; Weill Cornell Medicine, New York, New York, United States; Florida State University, Tallahassee, Florida, United States; Florida State University, Tallahassee, Florida, United States; Florida State University, Tallahassee, Florida, United States; Weill Cornell Medicine, New York, New York, United States; Florida State University, Tallahassee, Florida, United States

## Abstract

The Adherence Promotion with Person-centered Technology (APPT) trial addresses adherence challenges in cognitive assessment and training by leveraging smart, tailored technologies. Early detection of cognitive decline is critical for advancing scientific understanding, improving clinical trial recruitment, and enabling early intervention. However, adherence to frequent at-home testing and behavioral interventions remains a significant barrier. Designing an effective adherence support system requires understanding the motivations that drive older adults to participate in research and engage with interventions. To initially explore these motivations, we surveyed 472 older adults about their reasons for participating in research.

**Results identified four motivation typologies:**

brain health advocates, research helpers, fun seekers, and multiple motivation enthusiasts. Individual differences—including age, employment status, and cognitive difficulties—shaped motivation profiles, informing the development of tailored engagement strategies. Building on this foundation, we then pilot-tested a just-in-time adherence support system using personalized text messages to encourage engagement in a 10-day cognitive training study. Forty-three older adults received messages aligned with their stated participation motivations. Matched messages were rated as significantly more effective in promoting adherence than mismatched ones. Participants preferred messages that were personalized and formal, reinforcing the importance of message tailoring. With these insights, the first randomized controlled trial (N = 199) evaluating the APPT system for home-based cognitive training has been completed, and a second trial, examining home-based assessment, is now underway. Findings will inform future refinements of dynamic, machine-learning-driven adherence support, with broad applications for cognitive training, telehealth, and other health-related interventions.

